# HIV viral resuppression following an elevated viral load: a systematic review and meta‐analysis

**DOI:** 10.1002/jia2.25415

**Published:** 2019-11-20

**Authors:** Nathan Ford, Catherine Orrell, Zara Shubber, Tsitsi Apollo, Lara Vojnov

**Affiliations:** ^1^ Department of HIV World Health Organization Geneva Switzerland; ^2^ Centre for Infectious Disease Epidemiology and Research School of Public Health and Family Medicine University of Cape Town Cape Town South Africa; ^3^ Desmond Tutu HIV Centre Institute of Infectious Disease and Molecular Medicine Cape Town South Africa; ^4^ Department of Medicine University of Cape Town Cape Town South Africa; ^5^ Department of Infectious Disease Epidemiology Imperial College London London United Kingdom; ^6^ Government of Zimbabwe Ministry of Health and Child Care, AIDS and TB Unit Harare Zimbabwe

**Keywords:** adherence, enhanced adherence counselling, elevated viral load, viral suppression, viral resuppression

## Abstract

**Introduction:**

Guidelines for antiretroviral therapy recommend enhanced adherence counselling be provided to individuals with an initial elevated viral load before making a decision whether to switch antiretroviral regimen. We undertook this systematic review to estimate the proportion of patients with an initial elevated viral load who resuppress following enhanced adherence counselling.

**Methods:**

Two databases and two conference abstract sites were searched from January 2012 to October 2019 for studies reporting the number of patients with an elevated viral load whose viral load was undetectable when subsequently assessed. Data were pooled using random effects meta‐analysis.

**Results:**

Fifty‐eight studies reported outcomes of 45,720 viraemic patients, mostly from Africa (48 studies), and among patients on first‐line antiretroviral therapy (43 studies). Almost half (46.1%, 95% CI 42.6% to 49.5%) of patients with an initial elevated viral load resuppressed following an enhanced adherence intervention. Of those on first‐line ART with confirmed virological failure (6280 patients, 21 studies), only 53.4% (40.1% to 66.8%) were appropriately switched to a different regimen. Resuppression was higher among studies that provided details of adherence support. The proportion resuppressing was lower among children (31.2%, 21.1% to 41.3%) and adolescents (40.4%, 15.7% to 65.2%) compared to adults (50.4%, 42.6% to 58.3%). No important differences were observed by date of study publication, gender, viral failure threshold, publication status, time between viral loads or treatment regimen. Information on resistance testing among people with an elevated viral load was inconsistently reported.

**Conclusions:**

The findings of this review suggest that in settings with limited resources, current guideline recommendations to provide enhanced adherence counselling can result in resuppression of a substantial number of these patients, avoiding unnecessary drug regimen changes. Appropriate action on viral load results is limited across a range of settings, highlighting the importance of viral load cascade analyses to identify gaps and focus quality improvement to ensure that action is taken on the results of viral load testing.

## 1 Introduction

Guidelines for antiretroviral therapy in resource‐limited settings recommend enhanced adherence counselling following a first elevated viral load 1. This approach helps programme managers and clinicians to discriminate between suboptimal adherence and viral resistance as two possible reasons for elevated viral loads and guide an appropriate response in the absence of other information such as the results of drug resistance testing.

Several studies have highlighted the practical challenges of applying this viral load algorithm. Key challenges include ensuring that patients with an elevated viral load receive enhanced adherence counselling and a follow‐up viral load, and those whose viral load remains elevated are switched to a new antiretroviral regimen 2, 3. This has led to proposals to include HIV drug resistance testing as part of the algorithm^4^, or to switch all patients following an initial elevated viral load 5. Nevertheless, it is important from a patient and programme management perspective to avoid the additional cost and complexity of switching treatment regimens in individuals with an elevated viral load if the underlying reason is suboptimal adherence.

A review published in 2013 found that the majority of patients (71%) with an elevated viral load were subsequently found to have resuppressed following an adherence intervention. We undertook this systematic review to provide an updated estimate of the proportion of patients who resuppress following an initial elevated viral load.

## 2 Methods

This study has been designed and reported according to the Preferred Reporting Items for Systematic Reviews and Meta‐Analysis (PRISMA) statement 6. The protocol for this study is available online (Supplementary appendix).

An initial search strategy was developed and refined iteratively by reviewing known missing studies to identify additional terms to include in the final search strategy. Two investigators (NF, ZS), independently searched MEDLINE via PubMed and Embase, from 1 January 2012 (the date of the prior search 7) to 1 April 2019; this search was updated in PubMed on 27 October 2019. Abstracts from two major HIV conferences (IAS and CROI) from 2016 to 2019 were reviewed in order to identify studies that have been recently completed but not yet published. We supplemented database searches by screening bibliographies of review articles and all included full‐text articles. The same investigators scanned all abstracts and full‐text articles and achieved consensus on final study inclusions. To be included, studies had to report the number of patients with an elevated viral load (as defined by the studies) whose viral load was undetectable when subsequently assessed.

Two reviewers working independently and in pairs (NF, ZS), extracted data in accordance with a predefined protocol using a pre‐defined extraction form. The following information was extracted: study setting, age, sex, drug regimen, first or second‐line antiretroviral therapy (as defined by the studies) counselling and other support provided, viral load threshold, and number of patients with an elevated viral load who subsequently resuppressed and the number switched to second‐line ART. Indicators of study quality were also extracted according to predefined criteria, and used to assess the certainty of the evidence 8.

We calculated proportions and corresponding 95% confidence intervals (95% CIs) for patients resuppressed, and pooled data after transformation through random‐effects meta‐analysis 9, 10. Because statistical tests for heterogeneity do not work well with pooled proportions, we assessed potential sources of heterogeneity through the following predefined subgroup analyses: age, gender, documentation of enhanced adherence counselling, first‐ or second‐line regimen, publication status (published manuscript or conference abstract) and threshold for determination of virological failure. We analysed all data with Stata version 14.0.

## 3 Results

**Figure 1:**
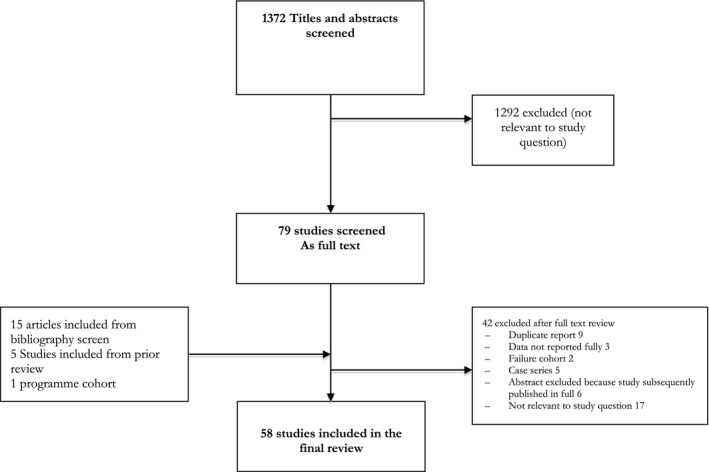
Study selection process.

Of the 1282 titles screened, 58 studies reporting outcomes of 45,720 viraemic patients met the inclusion criteria and were included in this review (Figure 1), including six studies from a prior review 7 Studies were conducted in 26 countries; most studies were conducted in South Africa (14 studies) and Uganda (12 studies); only three studies reported outcomes from high‐income settings 11, 12. Most studies were done among adults on first‐line ART (42 studies), and used a threshold of ≤ 1000 copies/mL to define viral suppression (33 studies). Thirty studies were reported as full articles, 28 as conference abstracts and there was one unpublished national HIV programme report (from Malawi) 13. Study characteristics are summarized in Table S1.

Methodological quality was considered to be moderate. Most studies were conducted retrospectively (36 studies) and almost half (28 studies) were available only as conference abstracts. Enhanced adherence counselling was documented for 37 studies, but details of adherence support were provided by only 14 studies. Only 13 studies carried out drug resistance testing on a sample with an elevated viral load. The certainty of the evidence was rated as low. Assessment of methodological quality is summarized in Table S2.

**Figure 2:**
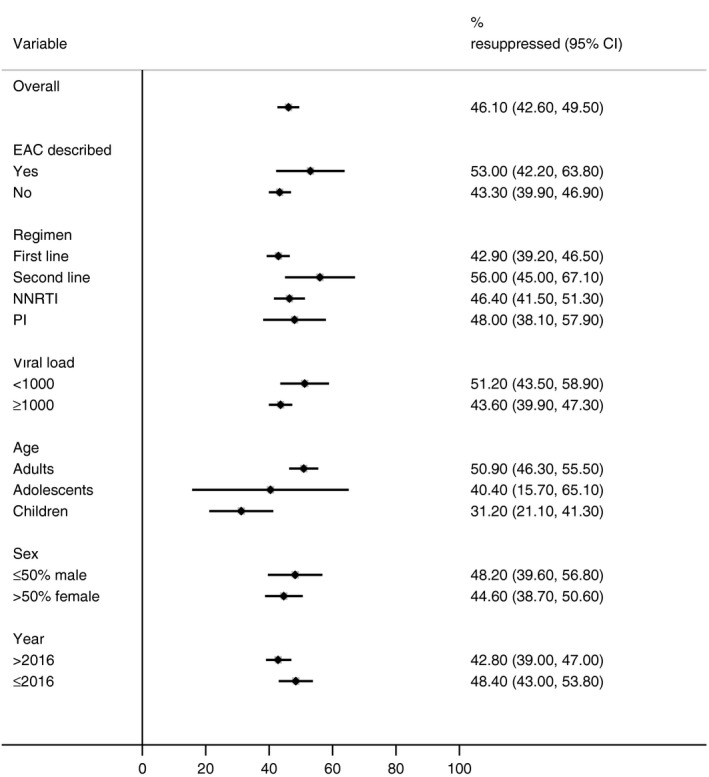
Proportion of patients with an elevated viral load who resupressed, by subgroup. EAC, Enhanced adherence counselling; NNRTI, non‐nucleoside reverse transcriptase inhibitor; PI, protease inhibitor.

Overall, almost half (46.1%, 95% CI 42.6% to 49.5%) of patients with an initially elevated viral load had resuppressed when viral load was subsequently measured; resuppression was higher among studies providing details of adherence support (53.0%, 42.2% to 63.8%) compared to studies where this was not described (43.3%, 39.9% to 46.9%) (Figure 2). The proportion of patients whose viral load was resuppressed was lower among children (31.2%, 21.1% to 41.3%) and adolescents 40.4%, 15.7% to 65.2%) compared to adults (50.9%, 46.3% to 55.5%) but numbers were small for these subgroup comparisons (652 children and 395 adolescents compared to 35,824 adults). No important differences were observed in other subgroup comparisons (date of study publication, gender, viral failure threshold, publication status, time between viral loads or treatment regimen.) (Figure 2).

The largest dataset was contributed by a routine programme report from Malawi (9803 patients with an initial elevated viral load) 13. The overall estimate for resuppression did not change importantly in a sensitivity analysis that dropped this programme report from the analysis (45.9%, 42.3% to 49.5%).

Twenty studies reported action taken for patients on first‐line ART in whom virological failure was confirmed (6352 patients); just over half of these patients (55.6%, 38.3% to 72.9%) were appropriately switched to second‐line ART. Information on resistance testing among people with an elevated viral load was inconsistently reported: only 11 studies reported data on resistance testing, which was only done on a sample of patients; however, as method of selecting patients for resistance testing was unclear, these data were not summarized.

Elements of enhanced adherence counselling varied across studies and countries. Patients with initial elevated viral loads were supported through frequent individual counselling sessions, depression and substance use screening, focus group discussions, and the provision of educational materials, cell phone and electronic reminder devices and pillboxes. However, only a minority of studies reported details of enhanced adherence counselling and approaches were too varied to be able to identify which specific approaches led to greater resuppression. Approaches provided and the corresponding proportion of patients who resuppressed are summarized in Table 1.

**Table 1:** Description of adherence interventions

Study	Who	What	Additional support	% Resuppressed (95% CI)
Billioux 14	Multi‐disciplinary team composed of physicians, clinic officers and counsellors	Additional one‐on‐one counselling with clinicians, assignment of a peer treatment supporter or in‐depth psychosocial counselling interventions by specialized staff at facility	Telephone follow up	59.8 (51.7 to 67.7)
Eholie 15	Trained health worker	Monthly therapeutic education sessions with the offer of up to nine adherence interventions	DOT by a relative; pill organizer; weekly phone call; daily alarm reminder; daily SMS; home visit; individual facility visit; peer group; rational prescribing to remove non‐essential meds	67.4 (60.3 to 73.9)
Etoori 16	Trained lay counsellors with involvement of nurses and psychologists for complicated cases.	Three counselling sessions over three months	Dedicated EAC register	58.9 (55.2 to 62.5)
Evans 17	Experienced adherence counsellor or social worker with specific training	Depression screen, alcohol and substance abuse evaluation; side effects of medications, social circumstances, employment status and depression indicators.	EAC: cell phone alarms and pillboxes Intervention study: Wisepill	83.9 (81.0 to 86.5)
Fox 18	Nurse and counsellor	One additional, intensive counselling session, and a second if abnormal results continue after session one	–	47.9 (44.5 to 51.3)
Garone 19	Medical officer or professional nurse	Adherence support: specific adherence barriers identified and assistance provided	–	67.1 (52.1 to 80.4)
Hoffmann 20	Trained adherence counsellors or nurses	Patient‐centred approach directed at identifying and overcoming specific barriers to adherence. One to two additional sessions	–	38.2 (34.9 to 41.5)
Jobanputra 21	Lay counsellor	Patients referred to a lay‐counsellor for three months’ counselling, then to a doctor if VL remains elevated.	–	54.0 (50.6 to 57.4)
Khan 22	Multidisciplinary team consisting of an HIV physician, psychologist, social worker and nurse	Structured, client‐centred adherence counselling	Pillboxes	61.5 (47.4 to 74.6)
Labhardt 23	Nurse/lay counsellor; village health worker (DOT)	Focus gp discussions, 1‐to‐1 counselling, DOT through village health worker	–	31.2 (23.2 to 39.9)
Lejone 2	Nurse/lay counsellor	Focus gp discussions, 1‐to‐1 counselling	–	27.0 (15.7 to 40.0)
Nasuuna 24	Nurse and adherence counsellor (including expert clients)	Monthly sessions to address barriers to adherence, social support, and opportunities to improve adherence using the 5 As (Assess, advise, assist, agree, arrange)	–	25.1 (20.4 to 30.1)
Navarro 12	Specialized nurse	Individualized behavioural intervention; community‐based social support services	Alarms; telephone follow up	50.4 (41.6 to 59.2)
Orrell 25	Local peer counsellor	Three educational sessions, and weekly counsellor home visits.	Pill boxes, dosing diaries	44.3 (30.1 to 59.0)
Wilson 26	Nurse of counsellor	Educational materials and counselling protocol	–	91.4 (82.3 to 97.4)

## 4 Discussion

This systematic review found that around half of patients with an initial elevated viral load had a suppressed viral load at subsequent measurement. These findings confirm that, in the absence of genotyping, enhanced adherence counselling helps to avoid unnecessary switching of ART in patients whose elevated viral load is due to suboptimal adherence.

The proportion of patients whose viral load was resuppressed was higher for those studies in which enhanced adherence counselling was documented. Resuppression following enhanced adherence counselling varied according to type and intensity of adherence support. One study, from eSwathini, found that in logistic regression the odds of resuppression increased when additional enhanced adherence counselling sessions was provided, compared to no intervention 16. A study from Zimbabwe found that patients undergoing three sessions of enhanced adherence counselling were more likely to resuppress compared to non‐attendance to enhanced counselling (relative risk of resuppression 2.8, 95% CI 1.8 to 4.2) 27. A study from South Africa found only a small benefit to EAC in terms of viral suppression within three months (8.1% increase compared to those not receiving EAC in adjusted analysis), and this difference was not sustained over 12 months 18.

Timeliness of EAC varied between studies. In one study from Uganda, in which only 25% of patients resuppressed, over half of patients received their first session more than six months after an elevated viral load was first detected 24 Longer time on first‐line ART has been found to be associated with a lower likelihood of resuppression 28; this is likely related to the observation that resuppression rates are lower in patients on first‐line ART in whom antiretroviral drug resistance mutations were subsequently detected 29. This review found higher rates of resuppression among patients on second‐line ART, consistent with the finding from Zimbabwe that being on second‐line ART at the time of initial high viral load test was associated with a higher probability of viral resuppression 27. The overall resuppression estimate from this meta‐analysis should therefore be considered a conservative estimate of the proportion of patients who could resuppress following adequate enhanced adherence support, given that the quality and approach to providing enhanced adherence support varied across studies and was not consistently reported.

Without drug resistance testing, it is difficult to determine with certainty whether elevated viral loads are the result of poor adherence or drug resistance. Due to high costs and complexity, viral load is used as a proxy indicator of treatment failure; however, it is important to ensure quality enhanced adherence counselling after an initial elevated viral load to more confidently ensure that patients with a second elevated viral load are switched to an alternative regimen on the assumption of acquired drug resistance. In this review, studies that provided details of enhanced adherence counselling tended to have higher rates of resuppression, which possibly reflects greater support provided during enhanced counselling. A study from several countries in West Africa assessed the effectiveness of an intensive adherence counseling intervention in patients with second‐line ART failure. In this study, 67% of patients (130/193) achieved viral resuppression; post study genotype resistance testing on stored samples found that the decision made based on enhanced adherence counseling alone was appropriate in 75% (145) of patients (15). Further studies are needed to help define the components of effective enhanced adherence counselling.

This review also found that only around half of patients on first‐line ART with virological failure confirmed by at least two viral load tests were switched to an alternative regimen. Several studies included in this review documented failure to take action at each step in the viral load cascade from initial viral load test, to adherence counselling and repeat testing, and regimen change in the case of persistent high viral load 23, 30, 31; these studies highlight the value of viral load cascade analyses to identify gaps and focus quality improvement to ensure that action is taken on the results of viral load testing.

Strengths of this review include a broad search to identify both published studies and conference abstracts, allowing for inclusion of data from over 45,000 patients with an initial elevated viral load. This expands the dataset considerably compared to the previous systematic review; this prior review found a higher proportion of patients resuppressing compared to the current review (70%, 57% to 84%), but this was based on just five studies contributing 406 patients to the analysis 7. The expanded dataset contributing to this current review allowed for exploration of the potential influence of factors such as treatment regimen, age and viral failure threshold. Limitations of the evidence base include study quality and incompleteness of reported information, and these provide several directions for research. In particular, the components of enhanced adherence counselling were inconsistently reported, and information about location (at facility or at home), duration and frequency was lacking. Limitations of study quality include retrospective study designs and a limited number of geographical regions contributing data, raising concerns about bias and generalizability of the findings. Few studies reported outcomes of patients tested for viral load, including numbers switching regimen in case of confirmed virological failure, loss to follow‐up and adverse clinical outcomes. Future studies are encouraged to report outcomes of viral load testing and provide a description of the components of enhanced adherence including the timing of the intervention, and drug resistance testing on patients unable to suppress following enhanced adherence to determine if non‐suppression is caused by drug resistance. Future research could also help to identify readily available information such as treatment history, duration on therapy or history of treatment interruptions that may be risk factors for non‐suppression to prioritize interventions 21.

## 5 Conclusions

In conclusion, the findings of this review suggest that in settings with limited resources and restricted access to drug resistance testing, current guidance to provide enhanced adherence counselling and support to patients presenting with a first elevated viral load can result in resuppression of a substantial number of these patients, avoiding unnecessary drug regimen changes.

The recommendation to first provided enhanced adherence support before deciding to switch treatment regimen is all the more relevant in settings adopting dolutegravir as part of first‐line antiretroviral therapy as the likelihood that high viral load is a consequence of suboptimal adherence rather than drug resistance is high, given the low levels of dolutegravir resistance.

## Competing interests

The authors declare that they have no conflicts of interest to disclose.

## Author’s contributions

NF and ZS designed the study, extracted and analysed the data. LV and CO contributed to the interpretation of the data. NF wrote the first draft of the manuscript which was reviewed, improved and approved by all authors prior to publication.

## Funding

This review was supported by funds from the Bill & Melinda Gates Foundation.

## Supporting information


**Table S1.** Characteristics of included studies

**Table S2.** Risk of bias

**Data S1.** Systematic review protocol
