# Supplementary material for: HIV viral resuppression following an elevated viral load: a systematic review and meta‐analysis
Source: J Int AIDS Soc. 2019 Nov 20;22(11):e25415. doi: 10.1002/jia2.25415 (PMC6864498; doi:10.1002/jia2.25415)
Supplement: Supplementary file 1 — Table S1. Characteristics of included studies [file JIA2-22-e25415-s001.docx]

**Table S1: Characteristics of included studies**

| **Study** | **Publication** | **Country** | **Regimen** | **Main drug/class** | **Population** | **% male** | **VL threshold** | **Time on ART** |
| --- | --- | --- | --- | --- | --- | --- | --- | --- |
| Bernheimer^1^ | Abstract | South Africa | Mixed | AZT | Children | 54% | 400 | 4 years |
| Billioux^2^ | Article | Uganda | 1st | . | Adults |  | 400 | 48 weeks |
| Breton^3^ | Abstract | Burundi, Cameroon, Côte d’Ivoire and Guinea | 1st | . | Adults | 39% | 1000 | 4 years |
| Bulage^4^ | Article | Uganda | 1st | EFV | All | 36% | 1000 | 80%>36M |
| Bvochora^5^ | Article | Zimbabwe | 1st | EFV | Adults | 45% | 1000 | 2-10 years |
| Calmy^6^ | Article | South Africa | 1st | NNRTI | Adults | . | 400 | . |
| Cassidy^7^ | Abstract | South Africa | 1st | ATV/r | Children | 53% | 400 | . |
| Castelnuovo^8^ | Abstract | Uganda | 1st | EFV | Adults | 39% | 1000 | 41 months |
| Chawana^9^ | Abstract | Zimbabwe | 2nd | . | Adolescents | 33% | 1000 | 19.4 months |
| Childs^10^ | Article | UK and Ireland | 1st | LPV/r | Children | 49% | 1000 | 21 months |
| Coffey^11^ | Article | USA | 1^st^ | DTG | Adults | 92% | 200 | 12 months |
| Devaux^12^ | Abstract | Burkina Faso, and Côte d'Ivoire | 1st | . | Children | . | 500 | 13 months |
| Dhodho^13^ | Abstract | Multiple (SSA) | 1st | EFV | Adults | . | 1000 | . |
| Etoori^14^ | Article | Eswatini | 1st | . | Adults | 35% | 1000 | 35 months |
| Euvrard^15^ | Abstract | South Africa | 1st | LPV/r | Adults | 30% | 1000 | 29 months |
| Evans^16^ | Article | South Africa | 2nd | . | Adults | 30% | 400 | . |
| Finci^17^ | Abstract | Mozambique | 2nd | . | Adults | 37% | 400 | . |
| Fox^18^ | Article | South Africa | 1st | LPV/r | Adults | 39% | 400 | . |
| Garone^19^ | Article | South Africa | 2nd | EFV | Adults | . | 1000 | 3.4 years |
| Giuliani^20^ | Abstract | Mozambique | 1st | ABC | All | 24% | 3000 | >6 months |
| Glass^21^ | Article | Lesotho | 1^st^ | EFV | Adults | 26% | 1000 | 36 months |
| Gupta^22^ | Article | Uganda | 1st | NNRTI | Adults | . | 1000 | 12 months |
| ^23^Hermans^24^ | Abstract | South Africa | 1st | EFV | Adults | . | 1000 | . |
| Hoffmann^25^ | Article | South Africa | 1st | . | Adults | 94% | 400 | . |
| Htung Naing^26^ | Abstract | Zimbabwe | 2nd | EFV | All | . | 1000 | . |
| Jahn^27^ | Unpublished | Malawi | 1st | EFV | Adults | . | 1000 | . |
| Jean Louis^28^ | Article | Haiti | 1st | . | All | 38% | 1000 | 39 months |
| Jobanputra^29^ | Article | Eswatini | 1st | . | All | 38% | 100 | 2.8 years |
| Kasimonje^30^ | Abstract | Zimbabwe | 1st | ATV/r | Adolescents | 42% | 50 | 6 years |
| Khan^31^ | Article | India | 2nd | EFV | Adults | 74% | 5000 | 7 years |
| Labhardt^32^ | Article | Lesotho | 1st | . | Adults | 34% | 80 | 4.1 years |
| Laxmeshwar^33^ | Abstract | India | 1st | NVP | Adults | 58% | 1000 | . |
| Lejone^34^ | Article | Lesotho | 1st | . | Children | 57% | 80 | 3.7 years |
| Mazzola^35^ | Abstract | Uganda | . | NVP | Adults | 35% | 1000 | 6.8 years |
| McCluskey^36^ | Article | Uganda | 1st | EFV | Adults | 36% | 1000 | 11 months |
| Mpawa^37^ | Article | Malawi - Prison | 1st | PI | Adults | 98% | 1000 | 32 months |
| Nanfuka^38^ | Abstract | Uganda | Mixed | NVP | Adults | 36% | 1000 | 6 years |
| Nasuuna^39^ | Article | Uganda | 1st | PI | Children | 47% | 1000 | 3.5 years |
| Navarro^40^ | Article | Spain | 2nd | . | Adults | 52% | 50 | NS |
| Nicholas^41^ | Abstract | Malawi | 1st | . | All | . | 1000 | . |
| Nicholas^42^ | Abstract | Uganda | 1st | . | . | . | 1000 | >6 months |
| Ntwali^43^ | Article | Rwanda | 1st | . | Adults | 33% | 1000 | . |
| Okoboi^44^ | Abstract | Uganda | 1st | EFV | Adults | 25% | . | 6.75 years |
| Orrell^45^ | Article | South Africa | 1st | EFV | Adults | 28% | 1000 | 189 days |
| Peterson^46^ | Article | Uganda & South Africa | 1st | EFV | Adults | 61% | 1000 | 16 months |
| Rutstein ^47^ | Article | Multiple | 1st | . | Adults | 53% | 1000 | . |
| Sher^48^ | Abstract | South Africa | . | . | Adolescents | 52% | 100 | >24 months |
| Simons^49^ | Abstract | Mozambique | 1st | . | All | . | 3000 | >6 months |
| Sproule^50^ | Abstract | Uganda | . | . | Adolescents | 48% | 1000 | 4.5 years |
| Sunpath^51^ | Abstract | South Africa | 1st | PI | Adults | . | 1000 | . |
| Tsondai^52^ | Abstract | South Africa | 1st | . | Adults | 0 | . | 31 months |
| van de Ven^53^ | Abstract | Tanzania | Mixed | . | All | . | 1000 | . |
| van Dijk^54^ | Abstract | Zimbabwe | 1st | . | . | . | 3000 | . |
| Venables^55^ | Abstract | South Africa | 1st | . | . | . | 1000 | . |
| Villa^56^ | Abstract | Ghana | 1st | NVP | Adults | 26% | 40 | 9 years |
| Wilson^57^ | Article | Thailand | 1st | ATV/r or LPV/r | Adults | . | 50 | 35 months |
| Zheng^58^ | Article | Multiple | 2nd | ATV/r or LPV/r | Adults | 57% | 400 | 39 weeks |

**References**

1. Bernheimer J, Patten G, Van Cutsem G, et al. Managing children and adolescents with HIV treatment failure: results from a pilot project in Khayelitsha. IAS, Vancouver, 2015. Abstract MoPeb204.

2. Billioux A, Nakigozi G, Newell K, et al. Durable Suppression of HIV-1 after Virologic Monitoring-Based Antiretroviral Adherence Counseling in Rakai, Uganda. *PLoS One* 2015; **10**(5): e0127235.

3. Breton G, Ndawinz J, Nimbona P, Sylla O, Akamba J-J, Mbangue Mea. HIV viral load monitoring in four West and Central African Countries: how is virological failure managed by caregivers in the OPP-ERA project ? *IAS 2017 Abstract MOPED1181*.

4. Bulage L, Ssewanyana I, Nankabirwa V, et al. Factors Associated with Virological Non-suppression among HIV-Positive Patients on Antiretroviral Therapy in Uganda, August 2014-July 2015. *BMC Infect Dis* 2017; **17**(1): 326.

5. Bvochora T, Satyanarayana S, Takarinda KC, et al. Enhanced adherence counselling and viral load suppression in HIV seropositive patients with an initial high viral load in Harare, Zimbabwe: Operational issues. *PLoS One* 2019; **14**(2): e0211326.

6. Calmy A, Ford N, Hirschel B, et al. HIV viral load monitoring in resource-limited regions: optional or necessary? . *Clin Infect Dis* 2007; **44:128–134**.

7. Cassidy T, O'Connell R, Makeleni T, de Azevedo V, Schulz T, Dumile Nea. Time to Switch? Outcomes of children and adolescents failing NNRTI regimens. *AIDS 2018 Abstract THPEB119*.

8. Castelnuovo B, Mubiru F, Nsumba M, Lwanga I, Lumu I, Kiragga Aea.

Compliance to guidelines for routine viral load testing in resource-limited settings. *Conference on Retroviruses and Opportunistic Infections 2018 Abstract 1130*.

9. Chawana T, Ngara B, Nathoo K, Katzenstein D, Nhachi C, Study A. An adherence intervention and drug resistance among HIV-positive adolescents failing second-line treatment at a public health clinic. *AIDS 2016 Abstract TUPEB096*.

10. Childs T, Shingadia D, Goodall R, et al. Outcomes after viral load rebound on first-line antiretroviraltreatment in children with HIV in the UK and Ireland: an observational cohort study. *Lancet HIV* 2015; **2**(4): e151-8.

11. Coffey S, Bacchetti P, Sachdev D, et al. RAPID antiretroviral therapy: high virologic suppression rates with immediate antiretroviral therapy initiation in a vulnerable urban clinic population. *AIDS* 2019; **33**(5): 825-32.

12. Devaux C, Toni T, Barry M, Desmonde S, Bosse C, Dahourou Dea. Virologic failure and drug resistance mutations patterns after 25 months on LPV/r-based antiretroviral therapy in children early treated before the age of two in West Africa *IAS 2017*.

13. Dhodho M, Frieden M, Shroufi A, Wanjiru W, Daho S, Simons E. Implementation of routine viral load monitoring in Lesotho, Malawi, Mozambique and Zimbabwe: a cascade analysis. *IAS 2017*.

14. Etoori D, Ciglenecki I, Ndlangamandla M, et al. Successes and challenges in optimizing the viral load cascade to improve antiretroviral therapy adherence and rationalize second-line switches in Swaziland. *JIAS* 2018; (21:e25194).

15. Euvrard J, Shroufi A, Osler M, Hilderbrand K, Trivino-Duran L, Boulle A. Resuppression after viraemia versus virologic failure in Khayelitsha, South Africa. *Conference on Retroviruses and Opportunistic Infections, Seattle, Washington, March 4–7, 2019 Abstract 1059*.

16. Evans D, Berhanu R, Moyo F, Nguweneza A, Long L, Fox M. Can Short-Term Use of Electronic Patient Adherence Monitoring Devices Improve Adherence in Patients Failing Second-Line Antiretroviral Therapy? Evidence from a Pilot Study in Johannesburg, South Africa. *AIDS Behav* 2016; **20:2717–2728**.

17. Finci I, Flores A, Gutierrez Zamudio A, de Abreu E, Issufo S, Gaspar Iea. The first experience with ART adherence clubs in Maputo, Mozambique: An analysis of a second-line ART subcohort. *AIDS 2018*

18. Fox M, Pascoe S, Huber A, et al. Effectiveness of interventions for unstable patients on antiretroviral therapy in South Africa: results of a cluster-randomised evaluation. 2018.

19. Garone D, Conradie K, Patten G, et al. High rate of virological resuppression among patients failing second-lineantiretroviral therapy following enhanced adherence support: A model of care in Khayelitsha, South Africa. *SAJHIVMED* 2013; **14(4):166-169.**

20. Giuliani R, Torrens A, Molfino L, Silva C, Ellman T, Magaia A. Viral load cascade and programmatic challenges after 2 years of routine HIV viral load testing in Maputo, Mozambique. *AIDS 2016*.

21. Glass TR, Motaboli L, Nsakala B, et al. The viral load monitoring cascade in a resource-limited setting: A prospective multicentre cohort study after introduction of routine viral load monitoring in rural Lesotho. *PLoS One* 2019; **14**(8): e0220337.

22. Gupta RK, Goodall RL, Ranopa M, et al. High rate of HIV resuppression after viral failure on first-line antiretroviral therapy in the absence of switch to second-line therapy. *Clin Infect Dis* 2014; **58**(7): 1023-6.

23. [http://www.differentiatedcare.org/Portals/0/adam/Content/yS6M-GKB5EWs_uTBHk1C1Q/File/Decision Framework.pdf](http://www.differentiatedcare.org/Portals/0/adam/Content/yS6M-GKB5EWs_uTBHk1C1Q/File/Decision%20Framework.pdf).

24. Hermans L, Tempelman H, Carmona S, Nijhuis M, Richman D, Grobbee Dea. High rates of viral resuppression on first line ART after initial virological failure. *Conference on Retroviruses and Opportunistic Infections 2018 Abstract 1118*.

25. Hoffmann CJ, Charalambous S, Grant AD, Morris L, Churchyard GJ, Chaisson RE. Durable HIV RNA resuppression after virologic failure while remaining on a first-line regimen: a cohort study. *Trop Med Int Health* 2014; **19**(2): 236-9.

26. Htung Naing Y, Bygrave H, Metcalf C, Munyaradzi D, Simons S, Bonyo Tea. Scaling up access to second line antiretroviral therapy in rural Zimbabwe: impact of routine viral load, model of care and re-suppression after switch. *AIDS 2015*.

27. Jahn A. Viral load monitoring cohort report Malawi (National) 2019.

28. Jean Louis F, Buteau J, Francois K, et al. Virologic outcome among patients receiving antiretroviral therapy at five hospitals in Haiti. *PLoS One* 2018; **13**(1): e0192077.

29. Jobanputra K, Parker LA, Azih C, et al. Factors associated with virological failure and suppression after enhanced adherence counselling, in children, adolescents and adults on antiretroviral therapy for HIV in Swaziland. *PLoS One* 2015; **10**(2): e0116144.

30. Kasimonje B, Shamu T, Chimbetete C. Experiences and outcomes of group psychotherapy as an antiretroviral adherence support intervention among young people failing on ART at Newlands Clinic, Harare, Zimbabwe. *AIDS 2018*.

31. Khan S, Das M, Andries A, et al. Second-line failure and first experience with third-line antiretroviral therapy in Mumbai, India. *Glob Health Action* 2014; **7**: 24861.

32. Labhardt ND, Ringera I, Lejone TI, et al. When patients fail UNAIDS' last 90 - the "failure cascade" beyond 90-90-90 in rural Lesotho, Southern Africa: a prospective cohort study. *J Int AIDS Soc* 2017; **20**(1): 21803.

33. Laxmeshwar C, Acharya S, Das M, Keskar P, Pazare A, Ingole Nea. Routine viral load testing and enhanced adherence counseling for ART monitoring at a public ART centre in Mumbai, India. *AIDS 2018*.

34. Lejone TI, Ringera I, Cheleboi M, et al. The Treatment Cascade in Children With Unsuppressed Viral Load-A Reality Check in Rural Lesotho, Southern Africa. *J Acquir Immune Defic Syndr* 2018; **77**(3): 250-6.

35. Mazzola R, Byawaka J, Birungi J, Sproule J, Nanfuka M, Mugisha Kea. Efficacy of intensive adherence counselling in reversing virologically-defined treatment failure in HIV-infected patients on long-term antiretroviral therapy in rural Uganda. *AIDS 2018*.

36. McCluskey SM, Boum Y, 2nd, Musinguzi N, et al. Brief Report: Appraising Viral Load Thresholds and Adherence Support Recommendations in the World Health Organization Guidelines for Detection and Management of Virologic Failure. *J Acquir Immune Defic Syndr* 2017; **76**(2): 183-7.

37. Mpawa H, Kwekwesa A, Amberbir A, et al. Virological outcomes of antiretroviral therapy in Zomba central prison, Malawi; a cross-sectional study. *J Int AIDS Soc* 2017; **20**(1): 21623.

38. Nanfuka M, Zhang W, Okoboi S, Birungi J, Kaleebu P, Tibengana Sea. Limited impact of first-line drug resistance mutations among patients receiving second-line therapy in Uganda. *IAS 2017*.

39. Nasuuna E, Kigozi J, Babirye L, Sewankambo N, Nakanjako D. Low HIV viral suppression rates following the intensive adherence counseling (IAC) program for children and adolescents with viral failure in public health facilities in Uganda. *BMC Public Health* 2018; **18:1048**.

40. Navarro J, Perez M, Curran A, et al. Impact of an adherence program to antiretroviral treatment on virologic response in a cohort of multitreated and poorly adherent HIV-infected patients in Spain. *AIDS Patient Care STDS* 2014; **28**(10): 537-42.

41. Nicholas S, Poulet E, Schramm B, Wapling J, Rakesh A, Amoros Iea. Increasing access to routine viral load with nearly point-of-care SAMBA-1: outcomes from a decentralised HIV program in Malawi. *AIDS 2016*.

42. Nicholas S, Schramm B, Poulet E, Ajule E, Candiru H, Adroa Pea. Viral load monitoring with SAMBA-1, a semi-quantitative nearly point-of-care method in Arua, a rural district in Uganda. *IAS 2017*.

43. Ndagijimana Ntwali JD, Decroo T, Ribakare M, et al. Viral load detection and management on first line ART in rural Rwanda. *BMC Infect Dis* 2019; **19**(1): 8.

44. Okoboi S, Chan K, Nanfuka M, Wangisi J, Nyonyitono M, Munderi Pea. Lack of effectiveness of adherence counselling on reversing virologic failure among long-term ART patients in rural Uganda. *AIDS 2014*.

45. Orrell C, Harling G, Lawn SD, et al. Conservation of first-line antiretroviral treatment regimen where therapeutic options are limited. *Antivir Ther* 2007; **12**(1): 83-8.

46. Petersen ML, Tran L, Geng EH, et al. Delayed switch of antiretroviral therapy after virologic failure associated with elevated mortality among HIV-infected adults in Africa. *AIDS* 2014; **28**(14): 2097-107.

47. Rutstein SE, Hosseinipour MC, Weinberger M, et al. Predicting resistance as indicator for need to switch from first-line antiretroviral therapy among patients with elevated viral loads: development of a risk score algorithm. *BMC Infect Dis* 2016; **16**: 280.

48. Sher R, Dlamini S, Muloiwa R. Patterns of detectable viral load in a cohort of HIV-infected adolescents on ART in Cape Town, South Africa. *AIDS 2018*.

49. Simons E, Ellman T, Giuliani R, et al. Role of community ART group (CAGs) in introducing routine HIV viral load monitoring in a rural district of Mozambique. *AIDS 2016*.

50. Sproule J, Byawaka J, Birungi J, Mazzola, R.S.A. , Nanfuka M, Moore Dea. Efficacy of intensive adherence counselling in reversing virologic failure in adolescents on long-term ART in Uganda. *AIDS 2018*.

51. Sunpath H, Naidu K, Pillay S, Moosa M, Dladla-Msimango P, Marconi V. The second cascade in the management of adult patients after first line ART failure in public HIV clinics in Durban, South Africa. *AIDS 2018*.

52. Tsondai P, Phillips T, Hsaio N, Petro G, Abrams E, Myer L. HIV viral load monitoring in HIV-infected pregnant women established on antiretroviral therapy in Cape Town, South Africa. *AIDS 2016*.

53. Van de Ven R, Antelman G, Haule D, van 't Pad Bosch J. Lessons from scaling-up HIV viral load testing in Tanzania: The importance of monitoring the HIV viral load cascade. *AIDS 2018*.

54. van Dijk J, Kamenova K, Shamu A, Pfeiffer K, Hobbins M, Ehmer J. Roll out of targeted viral load testing in two rural districts within Masvingo Province, Zimbabwe *AIDS 2016*.

55. Venables E, Giuliani R, Sebastiani T, Zhou J, Mazibuko B, Faniyan Oea. Viral load monitoring and second line therapy: do clinicians switch failing patients? *AIDS 2014*.

56. Villa G, Owusu D, Azumah M, Smith C, Abdullah A, Awuah Dea. Resuppression after point-of-care viral load testing to guide adherence counselling. *Conference on Retroviruses and Opportunistic Infections 2019 Abstract 1061*.

57. Wilson D, Keiluhu AK, Kogrum S, et al. HIV-1 viral load monitoring: an opportunity to reinforce treatment adherence in a resource-limited setting in Thailand. *Trans R Soc Trop Med Hyg* 2009; **103**(6): 601-6.

58. Zheng Y, Hughes MD, Lockman S, et al. Antiretroviral therapy and efficacy after virologic failure on first-line boosted protease inhibitor regimens. *Clin Infect Dis* 2014; **59**(6): 888-96.
