# Supplementary material for: HIV viral resuppression following an elevated viral load: a systematic review and meta‐analysis
Source: J Int AIDS Soc. 2019 Nov 20;22(11):e25415. doi: 10.1002/jia2.25415 (PMC6864498; doi:10.1002/jia2.25415)
Supplement: Supplementary file 2 — Table S2. Risk of bias [file JIA2-22-e25415-s002.docx]

**Table S2: Risk of bias**

| **Study** | **Published in full** | **Prospective** | **No selection** | **EAC documented** | **EAC completed** | **DR documented** |
| --- | --- | --- | --- | --- | --- | --- |
| Bernheimer | N | N | Y | Y | Unclear | N |
| Billioux | Y | N | N | Y | Y | N |
| Breton | N | N | Y | N | Unclear | N |
| Bulage | Y | N | Y | Unclear | Y | N |
| Bvochora | Y | N | Y | Y | Y | N |
| Calmy | Y | N | Y | Y | Y | N |
| Cassidy | N | Y | Y | Y | Unclear | N |
| Castelnuovo | Y | Y | Y | Y | Unclear | Y |
| Chawana | N | Y | Y | Y | Unclear | Y |
| Childs | Y | Y | N | N | Unclear | N |
| Coffey | Y | N | N | Y | N | Y |
| Devaux | N | Y | Y | N | Unclear | Y |
| Dhodho | N | N | Y | Y | Unclear | N |
| Etoori | Y | N | Y | Y | N | N |
| Eurvrard | N | N | Y | N | Unclear | N |
| Evans | Y | Y | Y | Y | Y | N |
| Finci | N | N | N | N | Unclear | N |
| Fox | Y | Y | Y | Y | Y | Y |
| Garone | Y | N | Y | Y | Y | Y |
| Giuliani | N | N | Y | Y | Unclear | N |
| Glass | Y | Y | Y | Y | Y | Y |
| Gupta | Y | Y | N | N | Unclear | Y |
| Hamers | Y | N | Y | N | Unclear | Y |
| Hermans | N | N | Y | N | Unclear | N |
| Hoffmann | Y | Y | Y | Y | Unclear | N |
| Htung Naing | N | N | Y | Y | Unclear | N |
| Jahn | N | N | Y | Y | Unclear | N |
| Jean Louis | Y | N | Y | Y | Unclear | N |
| Jobanputra | Y | N | Y | Y | N | N |
| Kasimonje | N | Y | Y | Y | N | N |
| Khan | Y | N | Y | Y | Y | N |
| Labhardt | Y | Y | Y | Y | Unclear | N |
| Laxmeshwar | N | N | Y | Y | Y | N |
| Lejone | Y | Y | Y | Y | Unclear | N |
| Mazzola | N | N | Y | Y | Y | N |
| McCluskey | Y | N | Y | N | Unclear | N |
| Mpawa | Y | Y | Y | Y | Unclear | N |
| Nanfuka | N | Y | N | N | Unclear | N |
| Nasuuna | Y | N | Y | Y | Y | N |
| Navarro | Y | Y | N | Y | Y | N |
| Nicholas | N | N | Y | N | Unclear | N |
| Nicholas | N | N | Y | N | Unclear | N |
| Ntwali | Y | N | Y | Y | Y | N |
| Okoboi | N | Y | Y | Y | Y | N |
| Orrell | Y | Y | Y | Y | Unclear | N |
| Peterson | Y | Y | N | N | N | N |
| Rutstein | Y | Y | Y | N | Unclear | Y |
| Sher | N | N | Y | N | Unclear | N |
| Simons | N | N | Y | Y | Unclear | N |
| Sproule | N | N | Y | Y | Y | N |
