# Supplementary material for: HIV viral resuppression following an elevated viral load: a systematic review and meta‐analysis
Source: J Int AIDS Soc. 2019 Nov 20;22(11):e25415. doi: 10.1002/jia2.25415 (PMC6864498; doi:10.1002/jia2.25415)
Supplement: Supplementary file 3 — Data S1. Systematic review protocol [file JIA2-22-e25415-s003.pdf]

Systematic review protocol

Resuppression following an initial high viral load: a systematic review and meta-analysis

1. INTRODUCTION

Since 2010, World Health Organization (WHO) Guidelines for antiretroviral therapy in resource-limited settings have proposed an algorithm for using viral load as a means for discriminating between patients in need of adherence support and those who require a switch to second-line therapy. A systematic review published in 2014 found that over two-third (71%) of patients with an initial high viral load (defined as a viral load >1000 copies/ml) had resuppressed following adherence counseling.[1]

Concerns have been expressed regarding the reliability of this approach, and that the current WHO algorithm may introduce unnecessary delays in patients failing treatment.[2] On the other hand, switching therapy after an initial high viral load risks unnecessarily placing non-adherent patients on more expensive, less tolerable second-line regimens.

The aim of this systematic review is to provide an updated assessment of the proportion of patients with an initial high viral load who resuppress following an adherence intervention, and identify possible risk factors for treatment failure associated with a single high viral load.

2. METHODS

2.1. Search strategy

An initial search strategy has been develope, and the list of titles was then cross-checked against known studies. Missing studies were then reviewed to identify additional terms to develop the final search strategy, which is detailed below.

Example search strategy for PubMed

#1	adherence
#2	Adherent
#3	adhere
#4	Compliance
#5	Compliant
#6	comply
**#7**	**#1 OR #2 OR #3 OR #4 OR #5 OR #6**

**#8**	**HIV Infections[MeSH] OR HIV[MeSH] OR hiv[tiab] OR hiv-1*[tiab] OR hiv-2*[tiab] OR hiv1[tiab] OR hiv2[tiab] OR hiv infect*[tiab] OR human immunodeficiency virus[tiab] OR human immunedeficiency virus[tiab] OR human immuno-deficiency virus[tiab] OR human immune-deficiency virus[tiab] OR ((human immun*[tiab]) AND (deficiency virus[tiab])) OR acquired immunodeficiency syndrome[tiab] OR acquired immunedeficiency syndrome[tiab] OR acquired immuno-deficiency syndrome[tiab] OR acquired immune-deficiency syndrome[tiab] OR ((acquired immun*[tiab]) AND (deficiency syndrome[tiab])) OR "sexually transmitted diseases, Viral"[MeSH:NoExp]**
**#9**	**Antiretroviral Therapy, Highly Active[MeSH] OR Anti-Retroviral Agents[MeSH] OR Antiviral Agents[MeSH:NoExp] OR ((anti) AND (hiv[tw])) OR antiretroviral*[tw] OR ((anti) AND (retroviral*[tw])) OR HAART[tw] OR ((anti) AND (acquired immunodeficiency[tw])) OR ((anti) AND (acquired immuno-deficiency[tw])) OR ((anti) AND (acquired immune-deficiency[tw])) OR ((anti) AND (acquired immun*) AND (deficiency[tw]))**
#10	viral load
#11	Virological
#12	Viraemia
#13	Viremia
#14	viraemic
#15	viremic
**#16**	**#10 OR #11 OR #12 OR #13 OR #14 OR #15**
#17	resuppression
#18	re-suppress
#19	resuppress
#20	suppression
#21	suppress
**#22**	**#17 OR #18 OR #19 OR #20 OR #21**
**#23**	**#7 AND #8 AND #9 AND #16 AND #22**

2.2. Databases

The following databases will be searched from November 01 2012 (the date of the last review) to present:

- Medline via Pubmed
- Embase

In addition, all Conferences of the International AIDS Society and the Conference on Retroviruses and Opportunistic Infections will be searched from 2016 onwards to identify studies that have been completed but not yet published as full text.

Finally, major treatment providers (MSF, ICAP, I-TECH, etc) will be contacted in an attempt to obtain unpublished programmatic information.

2.3. Restrictions
No language, or geographical exclusions will be applied.

2.4. Types of studies
- Randomized-controlled trials
- Prospective cohorts
- Retrospecitve cohort
- Programmatic data
- Case series <20 patients will be included

2.5. Types of participants

Inclusions
- Adults and children with a high initial viral load and at least one subsequent viral load

Exclusions
- Patients with only a single documented viral load

2.6. Types of outcomes

The following outcomes will be reported:
- Number/proportion with second viral load suppressed
- Number with clinical failure
- Number with documented drug resistance following initial high viral load

3.0. Risk of bias
Risk of bias will be assessed using appropriate tools for randomized trials and observational studies, adapted according to the final list of inclusions.

The risk of bias items assessed were as follows:

3.1. Randomized trials
- Method of randomization described
- Method of allocation concealment described
- Reasons for discontinuation provided
- No patient selection with respect to study inclusions
- No patient selection with respect to outcome reporting

3.2. Observational studies
- Prospective vs retrospective study design
- Adherence counseling documented
- Outcomes described for all patients

- Enhanced adherence counseling documented for all patients
- Drug resistance documented for all patients

4.0. Quantitative synthesis

Descriptive statistics will be used to compare the number of studies published over time. Point estimates and 95% confidence intervals will be calculated for the proportion of individuals resuppressing following an initial high viral load. Data will be pooled using the DerSimonian-Laird random-effects method,[3] with proportions transformed prior to pooling using the Freeman–Tukey double arcsine transformation[4] and then back-transformed to the original scale.[5] We will calculated the τ^2 statistic using DerSimonian and Laird method of moments estimator to assess between-study heterogeneity.[6]

Pre-planned subgroup analyses will be undertaken to determine the potential influence of the following covariates:

- Enhanced adherence counseling documented
- VL threshold
- Time to second VL
- Background drug resistance (if available)
- Age
- Advanced (CD4 <200) and very advanced (CD4 <100) HIV disease

All P values will be reported as two-sides and P<.05 considered significant. All analyses were conducted using Stata version 14.0 (StataCorp, College Station, Tex).

References

1. Bonner K, Mezochow A, Roberts T, Ford N, Cohn J. Viral load monitoring as a tool to reinforce adherence: a systematic review. *J Acquir Immune Defic Syndr* 2013; **64**(1): 74-8.
2. MSF. Making viral load routine Geneva: 2018.
3. DerSimonian R, Laird N. Meta-analysis in clinical trials. *Control Clin Trials* 1986; **7**(3): 177-88.
4. Freeman MF TJ. Transformations Related to the Angular and the Square Root. Annals of Mathematical Statistics 1950. 21: 607–611.
5. Miller J. The Inverse of the Freeman-Tukey Double Arcsine Transformation. The American Statistician. 1978. 32: 138.
6. Higgins JP, Thompson SG, Deeks JJ, Altman DG. Measuring inconsistency in meta-analyses. *Bmj* 2003; **327**(7414): 557-60.
